# TEMPORAL TREND OF HOSPITALIZATIONS FOR ACUTE BRONCHIOLITIS IN INFANTS UNDER ONE YEAR OF AGE IN BRAZIL BETWEEN 2008 AND 2015

**DOI:** 10.1590/1984-0462/2020/38/2018120

**Published:** 2019-11-25

**Authors:** Kanama Tumba, Talitha Comaru, Camila Machado, Manoel Ribeiro, Leonardo Araújo Pinto

**Affiliations:** a Pontifícia Universidade Católica do Rio Grande do Sul, Porto Alegre, RS, Brazil.

**Keywords:** Hospitalization, Bronchiolitis, Infant, Palivizumabe, Hospitalização, Bronquiolite, Lactentes, Palivizumabe

## Abstract

**Objective:**

To evaluate the trend of hospitalization for acute bronchiolitis in infants under one year of age, in the past eight years and after the implementation of the palivizumab immunization program in Brazil.

**Methods:**

The study is a retrospective analysis of data on infants younger than one year of age, who were hospitalized with acute bronchiolitis between 2008 and 2015 in Brazil. The Brazilian National Health System database was used. The rates of hospitalization in the pre-implementation (2008-2012) and post-implementation (2014-2015) periods of the palivizumab immunization program were evaluated. The total number of admissions in the same period was used as a comparison.

**Results:**

Between January 2008 and December 2015, 263,679 hospitalizations for bronchiolitis were recorded in infants younger than one year of age, 60% represented by boys. The incidence of hospitalization for bronchiolitis increased by 49% over this period (8.5 to 12.7 per 1,000 inhabitants per year). Between 2013 and 2014, the incidence rate of hospitalization for acute bronchiolitis decreased by 8% (12.5 to 11.5 per 1,000 inhabitants per year). However, in the second year of the program, hospitalization rate increased again by 10% (12.7 per 1,000 inhabitants per years).

**Conclusions:**

Acute bronchiolitis presented increasing rates of hospitalization over the study period. Hospitalization incidence for acute bronchiolitis declined one year after the implementation of palivizumab but increased again in the second year of the program.

## RESUMO

**Objetivo:**

Avaliar a tendência de hospitalização por bronquiolite aguda (BA) em lactentes menores de um ano de idade nos últimos oito anos no Brasil e, secundariamente, após a implementação do programa de imunização por palivizumabe.

**Métodos:**

Análise retrospectiva dos dados de lactentes menores de um ano de idade, hospitalizados com diagnóstico de BA entre 2008 e 2015 no Brasil, utilizando o banco de dados do Sistema Único de Saúde (SUS). Foram avaliadas as taxas de hospitalização nos períodos pré-implementação (2008-2012) e pós-implementação (2014-2015) do programa de imunização por palivizumabe. O número total de internações no mesmo período foi utilizado como comparação.

**Resultados:**

Entre janeiro de 2008 e dezembro 2015 foram registradas 263.679 internações por bronquiolite em lactentes menores de um ano de idade, 60% representado por meninos. A incidência de hospitalização por bronquiolite aumentou em 49% ao longo desse período (8,5 para 12,7 por mil ­habitantes/­ano). Entre 2013 e 2014, a taxa de incidência de hospitalização por BA diminuiu 8% (12,5 para 11,5 por mil habitantes/ano). Porém, no segundo ano do programa, a taxa de internação aumentou novamente em 10% (12,7 por mil habitantes/ano).

**Conclusões:**

A BA apresentou taxas de hospitalização crescente ao longo do período estudado. A incidência de hospitalizações de BA apresentou declínio um ano após a implementação de palivizumabe e retornou à tendência crescente no segundo ano do programa.

## INTRODUCTION

Acute bronchiolitis (AB) is the leading cause of hospitalization in emergency units and pediatric wards within patients’ first two years of life. It usually has a peak incidence between 2 and 6 months of age.^1^ ^,^ ^2^ Respiratory syncytial virus (RSV) is the etiological agent responsible for respiratory tract disease in most cases, and especially in the winter.^3^ ^,^ ^4^ ^,^ ^5^

Studies show that infants with risk factors such as prematurity, chronic lung disease, congenital heart diseases with hemodynamic instability, Down syndrome, or neuromuscular disease are more likely to develop severe RSV disease.^6^ ^,^ ^7^ The study by Sanchez-Luna et al. showed that intra-hospital mortality due to RSV from AB in children with pre-established risk factors was 18.8 times higher.^8^

In recent years, there has been a growing increase in hospitalizations for AB.^5^ ^,^ ^8^ ^,^ ^9^ In Brazil, data on hospitalization for AB are similar to worldwide reports.^10^ ^,^ ^11^ On the other hand, specific therapeutic management of RSV respiratory infection remains a challenge. Passive immunization through palivizumab, a humanized monoclonal antibody designed to prevent RSV respiratory infections for high-risk children, began in the United States in 1998 and showed a significant reduction in the rate of RSV hospitalizations.^12^ ^,^ ^13^ ^,^ ^14^ In Brazil, the Ministry of Health provided, through Ordinance No. 522, of May 2013, immunization through palivizumab for high-risk children nationwide: children under one year old that were born premature, in other words, they had a gestational age of less than or equal to 28 weeks; and children up to two years old with chronic lung disease or congenital heart disease with hemodynamic repercussion.^15^

In spite of our knowledge, we did not find a study that evaluated the incidence of hospitalization for AB in infants before and after the Ministry of Health introduced free palivizumab. Thus, the objective of the present study was to evaluate the temporal trend in the incidence of hospitalization for AB in children under one year of age, from 2008 to 2015, and to compare the periods before and after the implementation of the palivizumab immunization program in Brazil.

## METHOD

This is an ecological study based on a retrospective analysis of data from the Informatics Department of the Public Health System (*Departamentode Informática do Sistema Único de Saúde* - DATASUS). The information was obtained from the DATASUS website under the section “Informações de Saúde (TABNET)”. Because it is an open access platform, it was possible to access information about the absolute number of hospitalizations in the public health system, according to the International Classification of Diseases (ICD) version 10, characterized by the main diagnosis on admission to the hospital, which was verified in the section “Morbidade Hospitalar”. The absolute number of hospital admissions for AB in Brazil (ICD J21) in children under one year of age between January 2008 and December 2015 was evaluated. On this platform, there is no way to access clinical data, only the number of hospitalizations that can be stratified by range and location.

The variables were described in absolute and relative frequency. Based on these results, the incidence of hospitalization for AB was calculated by dividing the number of hospitalizations of children under one year of age by the population in the same age group, estimated annually by the Brazilian Institute of Geography and Statistics (*Instituto Brasileiro de Geografia e Estatística* - IBGE) during the study period, and multiplying the result by a thousand. We evaluated the trend in the incidence of hospitalization for AB throughout the study period, and before and after implementing palivizumab, in children under one year of age in Brazil.

The study was approved by the scientific committee of the institution where the analysis was performed. Considering that DATASUS is an open access data platform that does not have personal patient data, the study was exempt from making a submission to the Research Ethics Committee as it was an ecological study that did not access patient or individual data.

## RESULTS

Between January 2008 and December 2015, 4,536,266 hospitalizations were registered in Brazil, of which 263,679 occurred for AB in infants under one year of age, 60% of whom were male (Table 1).

**Table 1:** Number of hospitalizations from acute bronchiolitis, respiratory diseases, and all hospitalizations in children under one year of age in Brazil (2008-2015).

Year	Number of hospitalizations from AB (%)	Number of hospitalizations from respiratory diseases (%)*	Total number hospitalizations
2008	27,245 (4.8)	195,036 (34.8)	560,386
2009	29,608 (5.0)	210,761 (36.2)	581,804
2010	29,274 (5.1)	187,189 (32.8)	569,122
2011	34,900 (6.2)	184,972 (32.8)	562,229
2012	35,431 (6.3)	175,375 (31.3)	560,143
2013	37,053 (6.5)	174,912 (30.8)	566,278
2014	33,559 (5.9)	158,599 (28.2)	562,402
2015	36,593 (6.4)	155,970 (27.1)	573,902
Total	263,679 (5.7)	1,442,814 (31.8)	4,536,266

AB: acute bronchiolitis; *percentage related to all hospitalizations for respirtory diseases (ICD 10:J00-J99) in patients under one year of age.

**Figure 1:**
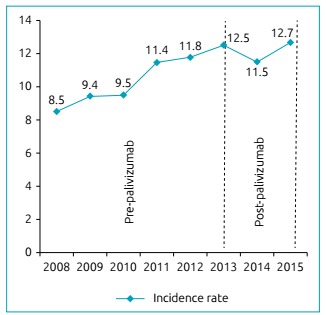
Coefficient of annual incidence of hospitalizations for acute bronchiolitis per thousand children/year in Brazil (2008-2015).

Hospitalizations for AB increased annually and corresponded to 5.8% of admissions for respiratory diseases in this age group during the analyzed period. Hospitalization rates for AB were found to gradually increase by 49%, from 8.5 per thousand inhabitants/year in 2008 to 12.7 per thousand ­inhabitants/­year in 2015 (Figure 1). Although the number of hospitalizations for AB has increased in the five administrative regions of the Brazilian territory, the cases are more concentrated in the South and Southeast Regions, where there was an annual proportion of cases of 67%.

The trend of hospitalizations for AB during this period continued to grow, except in 2014, the year following the implementation of passive immunization through palivizumab, when admissions dropped from 12.5 to 11.5 per 1,000 ­inhabitants/­year (down 8 %) among infants under one year of age throughout Brazil. However, in 2015, the second year of the program, it was observed that the hospitalization rate for AB returned to the level of 12.7 per thousand inhabitants/year (10% increase), as shown by Figure 1. The average annual incidence rate of AB throughout the period was 10.95 per 1000 inhabitants/year.

**Figure 2:**
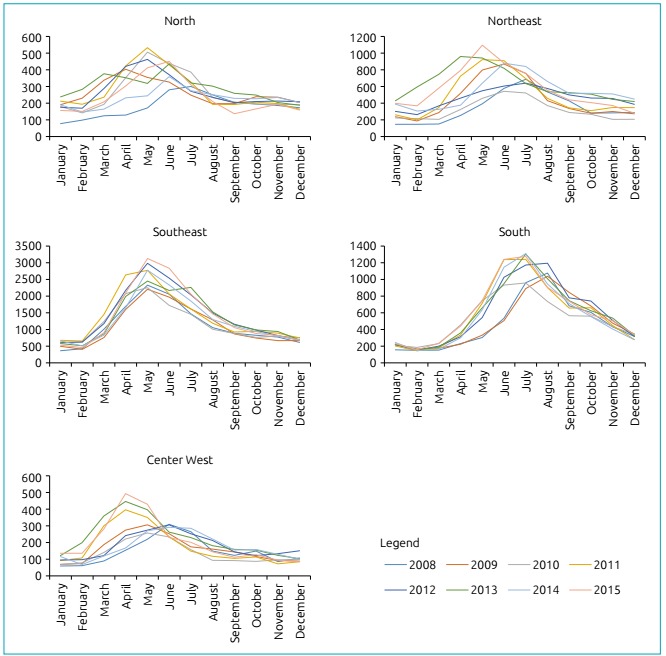
Monthly and annual distribution of hospitalizations for acute bronchiolitis in children under one year of age, by Brazilian region (2008-2015).

In the present study, seasonal variability was observed in relation to the beginning of the hospitalization period for AB in the different regions of Brazil (Figure 2). In the north and northeast regions, there was marked annual variation, with increased cases of AB, beginning, in some years, between January and February, with a peak number of hospitalizations between April and May. In the center west region, there was an abrupt onset from February, with hospitalization peaks in March or June, depending on the year. The southeast region has a clear annual variation, with a large number of cases from March and April, with a marked hospitalization peak in May. In the southern region, cases of AB started later, in March or April, with a peak of hospitalization between July and August.

## DISCUSSION

This study is the first to evaluate time trends in AB hospitalizations using data from across the national territory, before and after the introduction of the palivizumab immunization program. The results show a gradual increase in the incidence of hospitalizations for BA in all regions of Brazil over eight years, which are similar to studies conducted in industrialized countries.

In England, Green et al. showed that hospital admission rates for infants under one year of age that have AB, have steadily increased in recent decades (1979-2011).^16^ Leader et al., in the United States, also showed an increase in the hospitalization rate for AB in the first decade of 2000.^9^ Joanne Langley et al.’s study reported an increase in the incidence of hospitalizations for AB in Canadian children in the 1980-2000 period.^17^

The increased incidence of hospitalizations for AB may be multifactorial, including the increase in caesarean section and prematurity rates. Some authors associate the increase in AB with high prematurity rate due to a cesarean section before 37 weeks.^18^ ^,^ ^19^ These premature babies, in addition to developing incomplete lung function, are often subjected to ventilatory support and are at increased risk for respiratory problems. Additionally, preterm infants are predisposed to develop more severe viral infections of the lower respiratory tract, which require hospitalization. This could explain the increase in the hospitalization rate of AB during the first year of life in premature infants.

An overall estimate points to 135 million births in 2010. Among these, 15 million babies were born prematurely, representing an 11% rate of premature births. Brazil is among the ten countries with the highest prematurity rates, representing more than 10% of the births.^20^ The Ministry of Health, through the Health Information System, showed a 1.9-fold growth trend in premature births, ranging from 5% in 2005 to 11.8% in 2012.^21^

Regarding the number of cesarean sections, Brazil is listed among the ten countries with the highest number of premature births and, along with China, has maintained the rate of over 15% in recent years, above the recommendation of the World Health Organization (WHO).^20^ Recent data from the Ministry of Health’s Health Information System show a significant increase in cesarean section rates over the past ten years.^21^ ^,^ ^22^ The study performed by Silveira Santos et al. in 2008 showed that there was a significant increase in caesarean sections, representing 45% of all deliveries in Brazil.^23^

Finally, it is considered that caesarean sections and prematurity, together with urbanization of Brazil, may have contributed to the increased incidence of hospitalization for BA in recent years.

In our study, the southeast region had the highest proportion of hospitalizations due to its high population density, when compared to other administrative regions. Furthermore, our study showed that in Brazil, there is a different distribution of hospitalizations for AB in each region. According to Figure 2, hospitalizations occurred throughout the year, with different peak periods in each region, depending on the period of onset of seasonal variation. This result is consistent with data presented by the surveillance service to reinforce the importance of epidemiological studies of RSV infections and the passive immunization program in high-risk groups, such as premature infants and infants with bronchopulmonary dysplasia. Moreover, this result is also similar to the recent study by Freitas et al., which showed the seasonality of respiratory RSV infection in the five administrative regions of Brazil.^24^ Higher rates in the southern region, compared to the northeast region (which has a population larger than the Southern Region), demonstrate the possible relevant role of factors such as climate and behavior, especially the confinement associated with colder climates, which can lead to the spread of the virus.

The present study observed an 8% decline (from 12.5 to 11.5 per 1,000 inhabitants/year) in the hospitalization rate for AB among Brazilian infants one year after the implementation of the palivizumab passive immunization program (2013-2014). Increasing national accessibility of coverage can be considered to have contributed to the observed decline. Previously published clinical trials have shown better results using palivizumab in reducing the rate of hospitalization in high risk infants for severe lower respiratory tract disease from RSV.^12^ ^,^ ^13^ The study performed by Feltes et al. demonstrated for the first time in 1998 that palivizumab use reduced the risk of hospitalization and the development of severe RSV respiratory disease in high-risk infants.^25^ A study by Hasegawa et al., between 2000 and 2009, showed a significant decline in the incidence of AB hospitalizations due to RSV in premature children receiving palivizumab.26 The recent study performed by Doucette et al., which used nationally representative data from the United States, reported that there was a reduction in the incidence of AB from RSV hospitalization among infants with chronic lung disease over a 15-year period (1997-2012).^27^

In Brazil, in 2007, São Paulo was the first state to authorize the use of palivizumab during RSV season. In 2012, the Ministry of Health authorized the incorporation of palivizumab throughout Brazil for premature infants born with a gestational age of less than or equal to 28 weeks (up to 28 weeks and six days) younger than one year (up to 11 months and 29 days) and children under two years old (up to one year, 11 months, and 29 days) with pulmonary dysplasia or congenital heart disease with hemodynamic repercussion.^15^

The present study observed that in the second year of introducing palivizumab, the hospitalization rate for AB increased by about 10% (from 11.5 to 12.7 per 1,000 inhabitants/year). There are some plausible reasons for this trend in admission rates: the February 2015 revision of the Ministry of Health guidelines for a readjustment of immunobiological supply periods in different regions of the country, or the possible increase of another subgroup of pathological agents in addition to RSV.^14^ ^,^ ^28^

The present study had some limitations. First, it was a retrospective analysis, based on an open access data platform. Its objective was to estimate results in health services, whose reliability depended on the attending physicians making the correct diagnosis and properly inserting data when admitting cases. Second, this data platform cannot access patient records, making it impossible to analyze the subpopulation of premature patients. In addition, data in DATASUS are grouped into fixed age groups, and it is not possible to form subgroups from one month to six months and/or from six months to 12 months, which could provide further details on the data collected. In any case, the main objective was to describe the temporal and increasing tendency of AB.

Finally, AB presented increasing hospitalization rates over the study period. The incidence of AB hospitalizations declined one year after palivizumab was implemented, and they started to increase again in the second year of the program. Additionally, there is an important difference between the seasonality of AB in the different regions of Brazil

## References

[1] Carroll KN, Gebretsadik T, Griffin MR, Wu P, Dupont WD, Mitchel EF (2008). Increasing burden and risk factors for bronchiolitis-related medical visits in infants enrolled in a state health care insurance plan. Pediatrics.

[2] Jartti T, Lehtinen P, Vuorinen T, Ruuskanen O (2009). Bronchiolitis: age and previous wheezing episodes are linked to viral etiology and atopic characteristics. Pediatr Infect Dis J.

[3] Collins PL, Graham BS (2008). Viral and host factors in human respiratory syncytial virus pathogenesis. J Virol.

[4] Borchers AT, Chang C, Gershwin ME, Gershwin LJ (2013). Respiratory syncytial virus - a comprehensive review. Clin Rev Allergy Immunol.

[5] Stockman LJ, Curns AT, Anderson LJ, Fischer-Langley G (2012). Respiratory syncytial virus-associated hospitalizations among infants and young children in the United States, 1997-2006. Pediatr Infect Dis J.

[6] Sommer C, Resch B, Simões EA (2011). Risk factors for severe respiratory syncytial virus lower respiratory tract infection. Open Microbiol J.

[7] Murray J, Bottle A, Sharland M, Modi N, Aylin P, Majeed A (2014). Risk factors for hospital admission with RSV bronchiolitis in England: a population-based birth cohort study. PLoS One.

[8] Sanchez-Luna M, Elola FJ, Fernandez-Perez C, Bernal JL, Lopez-Pineda A (2016). Trends in respiratory syncytial virus bronchiolitis hospitalizations in children less than 1 year: 2004-2012. Curr Med Res Opin.

[9] Leader S, Kohlhase K (2003). Recent trends in severe respiratory syncytial virus (RSV) among US infants, 1997 to 2000. J Pediatr.

[10] Dall’Onder J, Lopes CL, Sechi FL, Sander MB, Eckert GU (2014). Profile of patients admitted in an intensive care unit for acute viral bronchiolitis in a South Brazilian children’s hospital. Rev AMRIGS.

[11] Straliotto SM, Siqueira MM, Muller RL, Fischer GB, Cunha ML, Nestor SM (2002). Viral etiology of acute respiratory infections among children in Porto Alegre, RS, Brazil. Rev Soc Bras Med Trop.

[12] The IMpact-RSV Study Group (1998). Palivizumab, a humanized respiratory syncytial virus monoclonal antibody, reduces hospitalization from respiratory syncytial virus infection in high-risk infants. Pediatrics.

[13] Ambrose CS, Chen X, Kumar VR (2014). A population-weighted, condition-adjusted estimate of palivizumab efficacy in preventing RSV-related hospitalizations among US high-risk children. Hum Vaccin Immunother.

[14] Yoshihara S, Kusuda S, Mochizuki H, Okada K, Nishima S, Simões EA (2013). Effect of palivizumab prophylaxis on subsequent recurrent wheezing in preterm infants. Pediatrics.

[15] Brasil. Ministério da Saúde. Secretaria de Ciência Tecnologia e Insumos Estratégicos, Departamento de Gestão e Incorporação de Tecnologias em Saúde (2012). Palivizumabe para a prevenção da infecção pelo vírus sincicial respiratório. Relatório de Recomendação da Comissão Nacional de Incorporação de Tecnologias no SUS - CONITEC - 16.

[16] Green CA, Yeates D, Goldacre A, Sande C, Parslow RC, McShane P (2016). Admission to hospital for bronchiolitis in England: trends over five decades, geographical variation and association with perinatal characteristics and subsequent asthma. Arch Dis Child.

[17] Langley JM, LeBlanc JC, Smith B, Wang EE (2003). Increasing incidence of hospitalization for bronchiolitis among Canadian children, 1980-2000. J Infect Dis.

[18] Kotecha SJ, Gallacher DJ, Kotecha S (2016). The respiratory consequences of early-term birth and delivery by caesarean sections. Paedriatr Respir Rev.

[19] Guimarães EA, Vieri CS, Nunes FD, Januário GD, Oliveira VC, Tubúrcio JD (2017). Prematurity and associated factores in divinopolis o Minas gerais state, Brazil 2008-2011: analysis of the information system on live birth. Epidemiol Serv Saude.

[20] World Health Organization (2012). Born Too Soon: the Global action report on preterm birth.

[21] Brasil. Ministério da Saúde - DATASUS (2012). Informações de saúde. Estatísticas Vitais. Nascidos vivos.

[22] Brasil. Ministério da Saúde. Agência Nacional de Saúde Suplementar (2016). Atualização das taxas de partos na saúde suplementar.

[23] Silveira MF, Santos IS, Barros AJ, Matijasevich A, Barros FC, Victora CG (2008). Increase in preterm births in Brazil: review of population-based studies. Rev Saude Publica.

[24] Freitas AR, Donalisio MR (2016). Respiratory syncytial virus seasonality in Brazil: implications for the immunisation policy for at-risk populations. Mem Inst Oswaldo Cruz.

[25] Feltes TF, Cabalka AK, Meissner HC, Piazza FM, Carlin DA, Top FH (2003). Palivizumab prophylaxis reduces hospitalization due to respiratory syncytial virus in young children with hemodynamically significant congenital heart disease. J Pediatr.

[26] Hasegawa K, Tsugawa Y, Brown DF, Mansbach JM, Camargo CA (2013). Trends in bronchiolitis hospitalizations in the United States, 2000-2009. Pediatrics.

[27] Doucette A, Jiang X, Fryzek J, Coalson J, McLaurin K, Ambrose CS (2016). Trends in Respiratory Syncytial Virus and Bronchiolitis Hospitalization Rates in High-Risk Infants in a United States Nationally Representative Database, 1997-2012. PLoS One.

[28] Brasil. Ministério da Saúde. Secretaria de Ciência Tecnologia e Insumos Estratégicos. Secretaria de Vigilância à Saúde (2015). Nota Técnica Conjunta 05/2015. Assunto: Estabelecer a sazonalidade do vírus sincicial respiratório no Brasil e oferecer esclarecimentos referentes ao protocolo do uso de Palivizumabe.

